# Bioinspired Adhesive and Antibacterial Microneedles for Versatile Transdermal Drug Delivery

**DOI:** 10.34133/2020/3672120

**Published:** 2020-05-08

**Authors:** Xiaoxuan Zhang, Guopu Chen, Yunru Yu, Lingyu Sun, Yuanjin Zhao

**Affiliations:** ^1^Department of Rheumatology and Immunology, The Affiliated Drum Tower Hospital of Nanjing University Medical School, Nanjing 210008, China; ^2^State Key Laboratory of Bioelectronics, School of Biological Science and Medical Engineering, Southeast University, Nanjing 210096, China

## Abstract

Microneedles have attracted increasing interest among various medical fields due to their painless, noninvasive, and efficient way of drug delivery. However, practical applications of these microneedles in different epidermal locations and environments are still restricted by their low adhesion and poor antimicrobial activity. Here, inspired by the antibacterial strategy of *Paenibacillus polymyxa* and adhesion mechanisms of mussel byssi and octopus tentacles, we develop hierarchical microneedles with multifunctional adhesive and antibacterial abilities. With polydopamine hydrogel as the microneedle base and a loop of suction-cup-structured concave chambers encircling each microneedle, the generated microneedles can fit the skin well; keep strong adhesion in dry, moist, and wet environments; and realize self-repair after being split into two parts. Besides, as polymyxin is loaded into both the hydrogel tips and the polydopamine base, the microneedles are endowed with excellent ability to resist common bacteria during storage and usage. We have demonstrated that these microneedles not only showed excellent adhesion when applied to knuckles and ideal antibacterial activity but also performed well in drug-sustained release and treatment for the osteoarthritis rat model. These results indicate that bioinspired multifunctional microneedles will break through the limitation of traditional methods and be ideal candidates for versatile transdermal drug delivery systems.

## 1. Introduction

Microneedles (MNs) could penetrate the skin without touching blood capillaries and nerve endings, thus providing a painless, noninvasive, and effective way for drug delivery [1, 2]. In recent years, various types of MNs have been developed, including silicon MNs [3], glass MNs [4], and hydrogel MNs [5, 6]. Among them, hydrogel MNs are playing an indispensable role because of their excellent biocompatibility, wide drug-carrying scope, and sustained drug release ability [7–11]. However, as most hydrogel MNs could not adjust well to the skin and would easily fall off from the skin, they highly rely on auxiliary equipment such as the medical adhesive tape to achieve ideal penetration and fixation. This poor adhesion ability seriously limits many practical applications of the hydrogel MNs, especially for the parts with high mobility and a large range of motion, such as joints. Besides, the biocompatible property of hydrogel MNs makes them extremely likely to be infected with bacteria [12–15], which not only causes inconvenience to the storage and usage but also increases the risk of skin infection. Therefore, hydrogel MNs with improved adhesion and antibacterial ability for drug delivery is highly desired.

**Figure 1:**
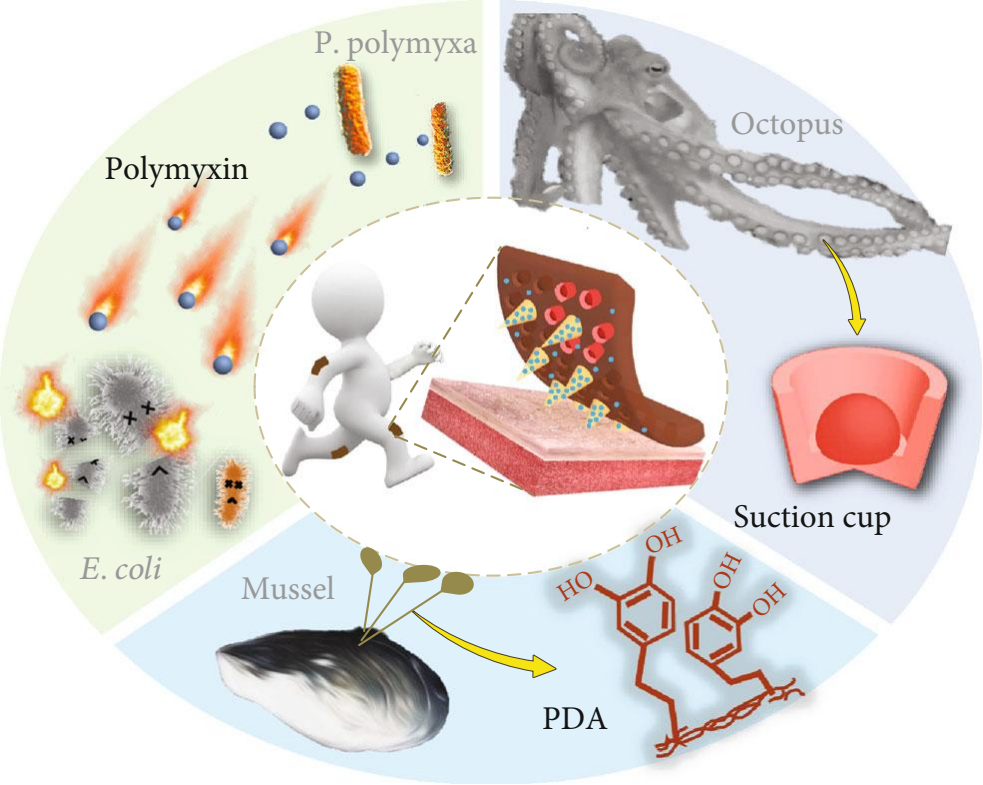
Schematic illustration of the bioinspired multifunctional MNs with adhesive and antibacterial abilities. The MNs have a mussel byssus-inspired PDA hydrogel as the base and are loaded with polymyxin derived from *P. polymyxa*. Each MN is surrounded with a circle of concave chambers, which mimic the suction cups of octopi.

In this paper, inspired by the adhesion features of mussels and octopi, together with the antibacterial mechanism of *Paenibacillus polymyxa* (*P. polymyxa*), we suggest novel MNs with desired functions for flexible biomedical applications, as presented in Figure 1. In nature, many organisms possess outstanding adhesion properties based on molecular attractions or microstructures of their surfaces. As a typical example of the former, mussels could stick to nearly all substrates tightly by the noncovalent and covalent chemical interactions between their adhesive protein of mussel byssi and substrates [16–19]. In comparison, octopi could strongly adhere to either dry or wet objects based on their suction cups and the inner dome-like protuberances [20, 21]. Additionally, *P. polymyxa* have been found to strikingly restrain the growth of other bacteria and even kill them by secreting a kind of antimicrobial peptide named polymyxin, which has a wide antimicrobial spectrum and could avoid drug resistance. These bioadhesive [22–26] and antibacterial [27–30] stratagems have aroused lots of inspiration among those working in the material and medical science fields. However, the introduction of any of these into the MN fields remains unexplored.

Herein, by integrating the molecular structure of adhesive protein of mussel byssi, the microstructure of octopus tentacles, and polymyxin from *P. polymyxa*, we present the desired multifunctional MNs for practical applications. Polydopamine (PDA) hydrogel was chosen as the flexible base of the MNs, which shared similar molecular structure to the adhesive protein and imparted the resultant MNs with strong adhesion. This integrated PDA could also help the MNs fit the skin and made them self-healing. Notably, each MN was surrounded by a circle of concave chambers with dome-like protuberances located inside. This octopus tentacle-mimicked structure further improved the adhesive ability, making the MNs adhesive in dry, moist, and wet environments. In addition, as the polymyxin was loaded into both the hydrogel tips and the PDA base, the MNs were endowed with the ability to resist common bacteria, such as *Escherichia coli* (*E. coli*). It was demonstrated that these MNs not only showed excellent adhesion when applied to knuckles and ideal antibacterial activity but also behaved well in drug-sustained delivery and treatment for the osteoarthritis rat model. This indicates that the bioinspired multifunctional MNs could break through the limitation of traditional methods which had difficulty in acting on the mobile parts effectively and provide a new and promising insight into the transdermal drug delivery of any part of the body.

## 2. Results

**Figure 2:**
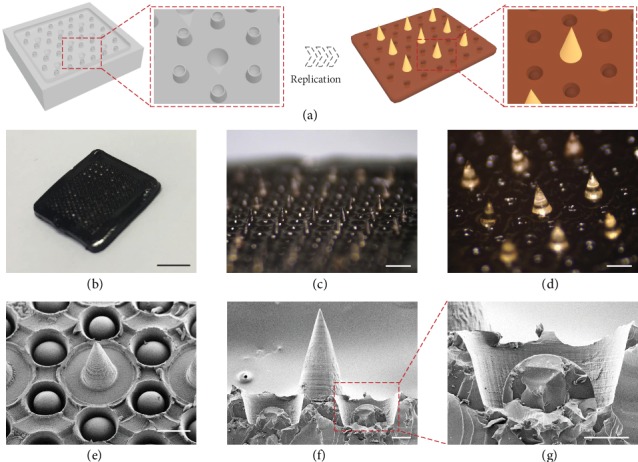
Fabrication and characterization of the bioinspired multifunctional MNs. (a) Schematic illustrations of the fabrication process. (b–d) Optical images of the MNs: an intact MN patch (b) and corresponding magnified images (c, d). (e–g) SEM images showing the suction-cup-structured concave chambers. The scale bars are 5 mm in (b), 1 mm in (c), 300 *μ*m in (d, e), and 100 *μ*m in (f, g).

In a typical experiment, the multifunctional MNs were prepared by replicating a specially designed negative mold (Figure 2(a)). The negative mold was composed of an ordered array of conical cavities, each of which was surrounded by six protuberances. It is worth mentioning that such a design could increase the density of the protuberances, and thus, the replicated MNs can have more suction cups and better adhesion ability. After adding PEGDA-sodium alginate solution to the negative mold as the MN tips and dopamine-gelatin mixture as the MN base and curing them successively, MNs with desired structure and adhesive property could be obtained (Figure 2(b)). The resultant MNs were found to replicate well the structure of the negative mold, with orderly aligned conical tips and their ambient concave chambers, as shown in Figures 2(c) and 2(d). In addition, it could be clearly seen from the scanning electron microscope (SEM) images that each concave chamber had a dome-like protuberance situated inside (Figures 2(e)–2(g)). Notably, this structure resembled well the suction cups of octopi, which also consisted of a single convex protuberance in each of the orifices.

The base of the fabricated MNs is a crosslinked hydrogel network formed by covalent bonding and noncovalent bonding of PDA and gelatin [31]. To be specific, when a small amount of sodium periodate was applied to the mixture of dopamine and gelatin, part of the dopamine would be instantly oxidized and then crosslinked to the gelatin backbone through the Schiff base reaction or Michael addition reaction; meanwhile, both the oxidized and unoxidized dopamine would assemble into stable PDA through hydrogen bonding, *π*-*π* stacking, etc. (Figure S1) All these interactions led to the gelation of the dopamine-gelatin mixture. Besides these types of physical and chemical bonding, the PDA hydrogel base possessed supramolecular interactions, imparting the MNs with self-healing ability. It was found that when the MN segments were put together, they could immediately weld together to form an integral MN again (Figure S2a). During this process, no external stimulus was applied, and the self-healed MN was strong enough to be held up (Figure S2b). In addition, similar to mussel byssi, the PDA hydrogel could tightly adhere to the skin depending on the various inner chemical groups, which endowed the MNs with ideal adhesion ability.

**Figure 3:**
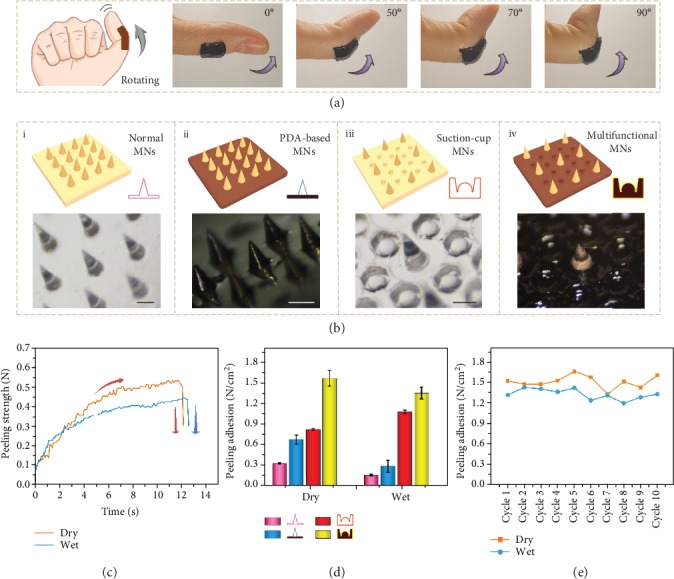
Measurement of adhesion ability. (a) Diagram and digital images of the bioinspired multifunctional MNs adhering to the knuckle of the thumb when the knuckle is bent to 90°. The thickness of the MNs was 2 mm. The scale bar is 1.5 cm. (b) Diagrams and optical images of four different MNs: normal MNs (i), PDA-based MNs (ii), suction-cup MNs (iii), and multifunctional MNs (iv). The scale bars are all 300 *μ*m. (c) Representative time-dependent profiles of peeling forces for multifunctional MNs in dry and wet conditions. (d) Dry and wet adhesion performances of the four different MNs. (e) Repeating cycles of the dry and wet adhesion performances of multifunctional MNs.

Combining the advantages of the PDA hydrogel and the suction-cup-like structure, the bioinspired multifunctional MNs exhibited desired adhesion ability in both dry and wet environments. To demonstrate it, these MNs were first applied to pork skin. Results showed that the MNs could strongly stick to the pork skin when the skin was lifted, bent, sprayed with water, or even immersed in water (Figure S3). The MNs were then attached to the knuckle of the thumb and were found to well fit the knuckle. As the thumb gradually flexed to 90°, the MNs bent correspondingly, indicating their excellent flexibility and adhesion ability (Figure 3(a)). Importantly, these abilities were retained after the MNs together with the thumb were sprayed with water, rinsed with water flow, or completely submerged in water (Figure S4). In addition, to further show the adhesion property, small weights were glued to the MNs one by one until they were detached from the thumb. It was demonstrated that the MNs could bear objects of 60 g, which was over 240 times their own mass (Figure S5).

Compared to their counterparts, the presented MNs exhibited improved adhesive performances under various conditions. To evaluate this, four different MNs were prepared for a peeling-off test. They were MNs without PDA hydrogel as the base or the suction-cup-like structure (normal MNs), MNs only with PDA hydrogel as the base (PDA-based MNs), MNs only with the suction-cup-like structure (suction-cup MNs), and MNs with both the PDA hydrogel as the base and the suction-cup-like structure (multifunctional MNs) (Figure 3(b)). During the test, the MNs with dissimilar features were first applied to a fixed substrate and then peeled off using customized equipment under either dry or wet environments (Figure S6). The peeling forces during the whole process were measured and recorded. A representative peeling force profile was a time-dependent curve with the quick rising, plateauing, and sudden plunging, as shown in Figure 3(c). It was demonstrated that normal MNs behaved the worst in both environments, while the multifunctional MNs performed the best (Figure 3(d)). As for the PDA-based MNs, although their peeling adhesion enhanced in dry environments in contrast to normal MNs, their performance was poor after contacting water. Besides, due to the rigid base, the overall peeling adhesion of the suction-cup MNs was inferior to the multifunctional MNs. Thus, by integrating the PDA hydrogel base and the suction-cup-like structure together, the bioinspired multifunctional MNs inherited their advantages as well as got rid of their drawbacks, possessing strong adhesion and broad application condition. Additionally, the adhesion ability of the multifunctional MNs was pretty stable, which remained almost unchanged during 10 consecutive cycles (Figure 3(e)).

**Figure 4:**
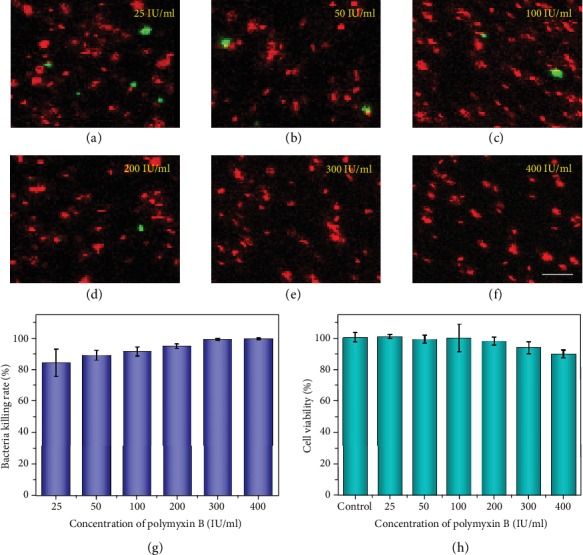
Antibacterial ability and cytocompatibility of multifunctional MNs at different polymyxin concentrations. (a–f) Confocal laser scanning images of fluorescence-stained *E. coli* in leaching liquor of multifunctional MNs at different original polymyxin concentrations. The scale bar is 5 *μ*m. (g–h) Statistical analysis of the *E. coli* killing rates (g) and NIH 3T3 cell viabilities (h) of multifunctional MNs at different original polymyxin concentrations.

In addition to adhesion properties, antibacterial activity was another necessary and important feature for MNs. This was because bacteria could not only enter into the skin via the microchannels generated by MNs but also adhere to biocompatible MNs, leading to infection and even severe complications. To impart the multifunctional MNs with antibacterial ability, polymyxin was loaded into the base and tips of the MNs to specifically resist and kill Gram-negative bacilli, which were the main types of bacteria that would cause infection. It was found that up to 80% of *E. coli* were killed by adding only a small quantity of polymyxin, and the killing ratios of *E. coli* increased corresponding to the increasing concentration of polymyxin (Figures 4(a)–4(f)). Notably, the killing rate reached 90% when the polymyxin concentration was more than 100 IU/ml (Figure 4(g)). However, the increasing concentration of polymyxin would inhibit the growth of normal cells. Thus, to find the optimal concentration, the MNs were immersed in the cell culture medium, and the leaching liquor was then used to culture NIH 3T3 cells. Results showed that the living ratios of the cells were over 85% in all the groups. Besides, the living ratios were close to 100% when the concentration of polymyxin was within 100 IU/ml, while they decreased after the concentration went beyond 100 IU/ml (Figure 4(h)). Based on all of these results, the appropriate concentration of polymyxin for both ideal cytocompatibility and desired antisepsis was set at 100 IU/ml.

Before the multifunctional MNs were used for in vivo disease treatment, their capacity for drug delivery was investigated. During this process, these MNs were first loaded with water-soluble small molecular drugs in their tips and then immersed in PBS solution to test their drug release kinetics. The experimental temperature was set as 37°C, which represented the body temperature. From the release curve, it could be seen that the MNs released drugs rapidly in the first 8 hours, then gradually slowed down until reaching the equilibrium after 2 days, which was about 80% of the total drug-loading amount (Figure S7). The results indicated that the MNs could realize ideal drug-sustained delivery with a suitable release rate and release amount.

**Figure 5:**
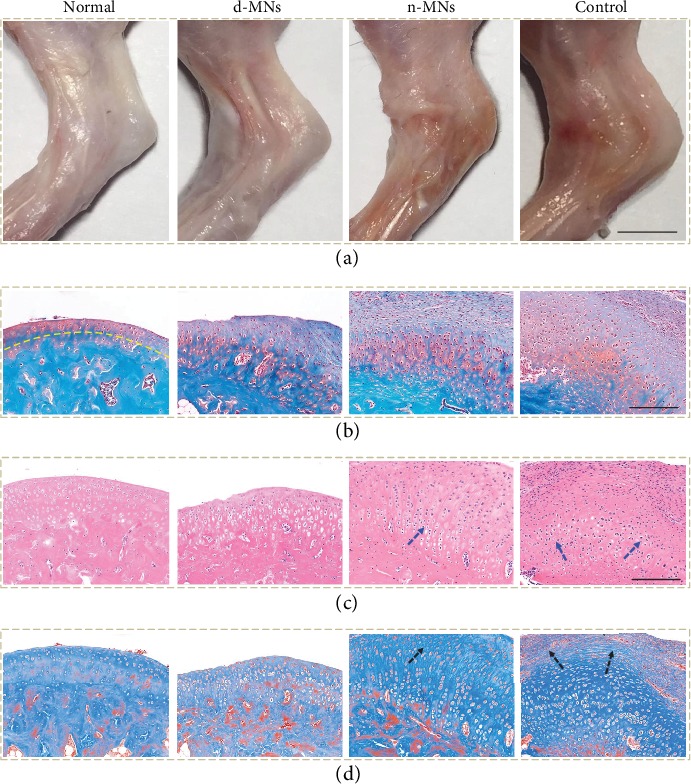
Evaluation of the multifunctional MNs on treatment for the KOA rat model. (a) Digital images of knee joints of rats in different treatment groups: the normal group, the d-MN group, the n-MN group, and the control group, respectively. (b) Corresponding Safranin O-fast green staining images. The yellow line indicates the tidemark. (c) Corresponding H&E staining images. The blue arrow indicates the disordered cartilage cells. (d) Corresponding Masson staining images. The black arrow indicates the fibrosis. The scale bars are 4 mm in (a) and 200 *μ*m in (b–d).

To demonstrate the practical performance of our MNs, MNs carrying glucocorticoid were fabricated and employed to treat a knee osteoarthritis (KOA) rat model. The KOA rat model was established by breaking the knee joints of SD rats in the overextension position (Figure S8). Except for the normal group as a blank control, other rats were equally divided into three groups: one group treated with glucocorticoid-loaded MNs (d-MN group), one group applied with unloaded MNs (n-MN group), and the last group without any treatment (control group). Compared to the d-MN group, the rat knee joints of the n-MN group and the control group displayed more severe joint lesions, as they were more inflamed, more swollen, stiffer, and extremely inflexible (Figure 5(a) and Figure S9). This result preliminarily verified the therapeutic effects of glucocorticoid-loaded MNs on KOA and illustrated that the physical stimulation with MNs alone was not effective in KOA treatment.

To further explore the curative effects of glucocorticoid-loaded MNs, tissue slices in each group were stained with Safranin O-fast green, hematoxylin-eosin (H&E), and Masson. It was seen from the Safranin O-fast green staining that in the n-MN group and the control group, the surfaces of cartilages were obviously uneven and the tidemarks were irregular and almost indiscernible. On the contrary, the d-MN group showed an evident recovery of such lesions (Figure 5(b)). Similarly, the results from the H&E and Masson staining further demonstrated the lesser disorder of the structure of cartilage cells, alleviated inflammatory cell infiltration, and reduced formation of cell clusters as well as decreased fibrosis of the d-MN group, relative to the n-MN group and the control group (Figures 5(c) and 5(d)). In addition, the Mankin scoring also revealed the better recovery of the d-MN group based on the structure change, cellular abnormalities, matrix staining, and tidemark variation (Figure S10). Therefore, transdermal drug delivery via MNs was believed to enhance drug penetration, promote drug absorption, and improve drug utilization, which has potential to replace traditional drug delivery methods for many other disease treatments.

## 3. Discussion

In summary, we have presented bioinspired adhesive and antibacterial MNs which can be employed in versatile transdermal drug delivery. Making MNs adhesive to skin and resistant to bacteria has been an ongoing issue in MN-related fields. The poor adhesion ability of most MNs requires their usage in the company of auxiliary equipment like a medical adhesive tape, which may bring about inconvenience in application and cause discomfort to skin regions. To improve their adhesion ability [32], attention has been mainly focused on chemically modifying the MN tip materials or choosing swellable hydrogels. However, their practical performances are still unsatisfactory owing to the complicated physiological environment of the skin. To our knowledge, designing microstructures of the MN base to improve their adhesion ability is seldom explored. Beyond that, the microchannels provided by biocompatible MNs have been reported to go through the skin and act as entry gates for bacteria to enter into the body, leading to bacterial infection or even life-threatening complications [33]. This problem could even be aggravated by the relatively long application time of MNs. Thus, to deal with this problem, MNs loaded with antibiotics or metal ions (e.g., Ag^+^) have been put forward to realize their self-sterilization. However, their drug resistance, allergenicity, and ion cytotoxicity still affect the practical performances. Therefore, more efforts are still anticipated to develop new adhesive, antibacterial MNs.

We have herein fabricated bioinspired multifunctional MNs with ideal adhesive and antimicrobial abilities to well solve the aforementioned problems. The adhesive PDA hydrogel base and suction-cup-mimicked microstructure impart the MNs with excellent adhesion ability, while the antimicrobial peptides from *P. polymyxa*, known for their low biological toxicity, wide antibacterial spectrum, high bacterial lethality, and no drug resistance, make the MNs bacteria-resistant and safe. The fabricated MNs also possess many other features, including flexibility, self-repair, and drug-sustained release. These characteristics enable our MNs to be used versatilely, including a long time and sustainably delivering drugs to highly mobile body parts (joints, hands, legs, etc.) and susceptible body regions (armpits, feet, face, etc.). Thus, it is believed that these MNs can be promising candidates in transdermal drug delivery devices, wearable biomedical systems, and so on.

## 4. Materials and Methods

### 4.1. Materials, Cell Lines, and Animals

Poly(ethylene glycol) diacrylate (PEGDA, average Mw is about 700), dopamine hydrochloride, gelatin, and rhodamine B (RhB, ≥95%, HPLC) were obtained from Sigma-Aldrich. Sodium alginate was bought from Alfa Aesar. Sodium periodate and 2-hydroxy-2-methylpropiophenone (HMPP) were purchased from Aladdin. Polymyxin B (10000 IU/ml) was provided by Hangwei Corp. Glucocorticoid was provided by Jinling Hospital. SYTO and propidium iodide (PI) were supplied by KeyGEN BioTECH Corp. The MTT reagent was obtained from J&K Scientific Ltd., Shanghai. Phosphate-buffered saline (PBS) was self-prepared in the laboratory. A Millipore Milli-Q system provided deionized water with a resistivity of 18.2 M*Ω*·cm^−1^. NIH 3T3 cell lines were purchased from the Cell Bank of the Chinese Academy of Sciences (Shanghai, China). Cells were cultured in high glucose Dulbecco's Modified Eagle's Medium (DMEM, Gibco, USA) with 10% (*v*/*v*) fetal bovine serum (FBS, Gibco, USA) and 1% (*v*/*v*) penicillin-streptomycin double antibiotics in the incubator (HERAcell 150, Thermo Scientific, USA) under 37°C, 5% CO_2_. The 280-320 g male Sprague Dawley (SD) rats were from Jinling Hospital. Animals were treated in strict accordance with the recommendations in the Guide for the Care and Use of Laboratory Animals of China. All the animal care and experimental protocols were reviewed and approved by the Animal Investigation Ethics Committee of Jinling Hospital.

### 4.2. Characterization

The optical images of MNs were observed by a stereomicroscope (JSZ6S, Jiangnan Novel Optics) and recorded by CCD (Oplenic Digital Camera). Digital images of the pork skin, the knuckle, and SD rats were captured by a digital camera (Canon 5D Mark II). The SEM images were characterized using a field scanning electron microscope (FESEM, UltraPlus, Zeiss). The fluorescence images were taken by a laser scanning confocal microscope (OLYMPUS, FV10i).

### 4.3. Fabrication of the Bioinspired Multifunctional MNs

PEGDA (50% *v*/*v*), HMPP (1% *v*/*v*), and sodium alginate (2% *w*/*v*) were first mixed together in the deionized water. 200 *μ*l of the mixed solution was then added to a custom-built negative mold and placed in a vacuum for 10 min to fully fill the cavities. After the redundant solution was sucked up using a pipettor, 450 *μ*l solution containing 0.0127 g sodium periodate, 0.0225 g dopamine hydrochloride, 0.075 g gelatin, and a small droplet of NaOH (1 M) was added to cover the negative mold. Polymyxin was premixed with the PEGDA solution and the dopamine solution with the final concentration of 25 IU/ml, 50 IU/ml, 100 IU/ml, 200 IU/ml, 300 IU/ml, or 400 IU/ml. The PEGDA-sodium alginate tips were solidified by UV irradiation (365 nm, 10 W) for 15 s, and the dopamine-gelatin base was gelated under room temperature for 20 min. Notably, due to its low power and short application time, the applied UV irradiation could also be applicable to biologics. Finally, the resultant MNs (300 *μ*m in diameter and 600 *μ*m in length) with orderly aligned concave chambers (375 *μ*m in the top diameter and 300 *μ*m in the bottom diameter) were gently peeled out of the negative mold.

### 4.4. Peeling-Off Test

Pork skin bought from the local butcher's was first cut into about 3 × 2 cm^2^ pieces, followed by being washed with deionized water. The dried skin pieces were then glued to the underlying bracket of an electronic tensile testing machine (HP-500, Handpi®). Four different kinds of MNs, normal MNs, PDA-based MNs, suction-cup MNs, and multifunctional MNs, were placed on the surface of the pork skin pieces and were pressed with the same force for 5 s. It should be mentioned that for variable control, the total number of microneedles and suction cups combined per unit area, as well as the size of four kinds of MNs, was kept the same. After that, the MNs were lifted off with the initial peeling angle of about 10° and the displacement velocity at about 0.1 mm/s. During this process, the time-dependent peeling strength was recorded by a bundled software (MulForce). The peeling adhesion was calculated by dividing the peeling strength before the detachment by the contact area.

### 4.5. Antibacterial Ability Test

The Gram-negative bacteria *E. coli* were chosen to investigate the antibacterial ability of the MNs. MNs containing different polymyxin concentrations, 25 IU/ml, 50 IU/ml, 100 IU/ml, 200 IU/ml, 300 IU/ml, and 400 IU/ml, were fabricated with the aforementioned method and immersed in PBS solution (pH = 7.4) for 2 days. Then, 1 ml of the leaching liquor of the MNs was mixed with 100 *μ*l of the *E. coli* solution (10^4^ CFU ml^−1^) and incubated in the 48-well plate for 6 h at 37°C. To visualize the bacteria viability, the bacteria were stained with two staining agents, SYTO for 20 min and PI for 5 min in the dark. As a result, the live *E. coli* were dyed green and dead ones were dyed red.

### 4.6. Biocompatibility Test

MNs containing different polymyxin concentrations were cultivated in the culture medium for 2 days. Subsequently, the leaching liquor was sterilized by 200 nm syringe filters and added into a 48-well plate (1 ml per well). 100 *μ*l of cell suspensions (about 5 × 10^4^ cells) was seeded into each well on the plate and cultured for 2 days. After that, 100 *μ*l MTT solution was added into the wells and incubated for 4 h. Finally, the entire medium was removed, and 500 *μ*l DMSO was added to totally dissolve the formazan crystals. The cell viability was obtained by reading out the OD values of the DMSO solutions using a microplate reader (Synergy HTX, BioTek, USA).

### 4.7. Drug-Sustained Release Test

RhB was employed as a model for water-soluble small molecular drugs. MNs containing 0.5 mg/ml RhB were immersed in PBS solution and shaken on a thermomixer (Eppendorf) at the rate of 450 r/min under 37°C. The time-varying OD values of the PBS solution were measured by the microplate reader (540 nm excitation wavelength). Based on the OD values and the standard curve of RhB, the release amount of RhB from the MNs could be calculated.

### 4.8. KOA Rat Model Establishment and Treatment

280-320 g male SD rats were first anesthetized by intraperitoneal injection of 10% chloral hydrate. The right knee joints of the rats were fixed by tubular plaster in the overextension position, and the plaster was wrapped with a plastic shell to prevent the rats biting the plaster. The right knee joints were immobilized for 3 weeks. During this period, the plaster was to be immediately adjusted or changed if it got loose, fell off, or caused skin necrosis and ulcers. The rats were then equally divided into three groups, which were applied with glucocorticoid- (10 mg/ml) loaded MNs, unloaded MNs, and no treatment. An applicator was employed to apply these MNs to the skin, which had a relatively weak attachment to the back of the MNs and could be detached from the MNs after application. The MNs were changed every day. It should be mentioned that during the removal, a small quantity of water was first applied to lower the adhesion of the PDA hydrogel. Then, starting from the edge, the MNs could be gently peeled off. After consecutive treatments for 7 days, the rats were sacrificed and their right knees were sampled. The joints were fixed in 4% paraformaldehyde, decalcified, embedded in paraffin, sliced up, and stained with Safranin O-fast green, hematoxylin-eosin (H&E), and Masson, respectively. The degrees of joint lesions were statistically analyzed using a modified Mankin scoring system [34, 35].

## Supplementary Materials

Supplementary Materials Figure S1: schematic illustration of the crosslinking of the PDA hydrogel base. Figure S2: self-repair of the multifunctional MNs. Figure S3: digital images showing that multifunctional MNs adhered to the pork skin when they were suspended and bent, in a moist environment and in water. Figure S4: digital images showing that multifunctional MNs adhered to the knuckle of the thumb when they were bent from 0° to 90°. Figure S5: loading test of multifunctional MNs. Figure S6: schematic illustration of the peeling-off test process. Figure S7: in vitro drug release of multifunctional MNs under 37°C. Figure S8: digital images showing the establishment of the KOA rat model. Figure S9: changes in swollen degree of the rat knees. Figure S10: grading of the severity of KOA lesion with the modified Mankin scoring system in different rat groups.
