# Supplementary material for: Bioinspired Adhesive and Antibacterial Microneedles for Versatile Transdermal Drug Delivery
Source: Research (Wash D C). 2020 May 8;2020:3672120. doi: 10.34133/2020/3672120 (PMC7231261; doi:10.34133/2020/3672120)
Supplement: Supplementary Materials — Figure S1: schematic illustration of the crosslinking of the PDA hydrogel base. Figure S2: self-repair of the multifunctional MNs. Figure S3: digital images showing that multifunctional MNs adhered to the pork skin when they were suspended and bent, in a moist environment and in water. Figure S4: digital images showing that multifunctional MNs adhered to the knuckle of the thumb when they were bent from 0° to 90°. Figure S5: loading test of multifunctional MNs. Figure S6: schematic illustration of the peeling-off test process. Figure S7: in vitro drug release of multifunctional MNs under 37°C. Figure S8: digital images showing the establishment of the KOA rat model. Figure S9: changes in swollen degree of the rat knees. Figure S10: grading of the severity of KOA lesion with the modified Mankin scoring system in different rat groups. [file 3672120.f1.docx]

Supplementary Materials for

**Bioinspired adhesive and antibacterial microneedles for versatile transdermal drug delivery**

Xiaoxuan Zhang, Guopu Chen, Yunru Yu, Lingyu Sun, Yuanjin Zhao*

*Corresponding author. Email: yjzhao@seu.edu.cn

**Supplemental Figures**


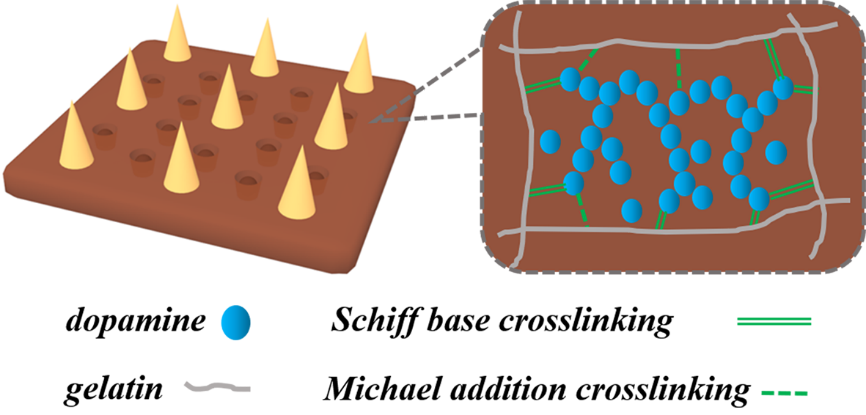


**Figure S1.** Schematic illustration of the crosslinking of the PDA hydrogel base.


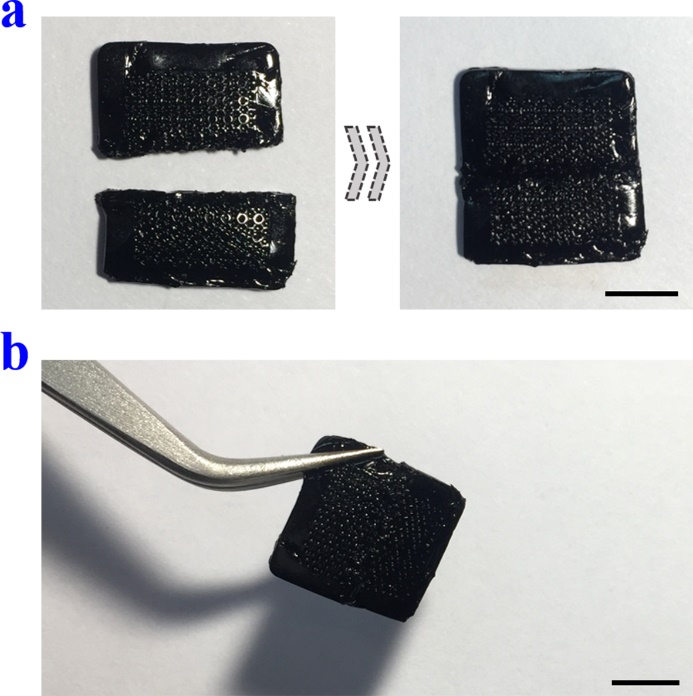


**Figure S2.** **Self-repair of the multifunctional MNs.** (a) Digital images of an integral MN formed by two cut MN pieces. (b) Digital image of the self-healed MN being held up. The scale bars are all 5 mm.


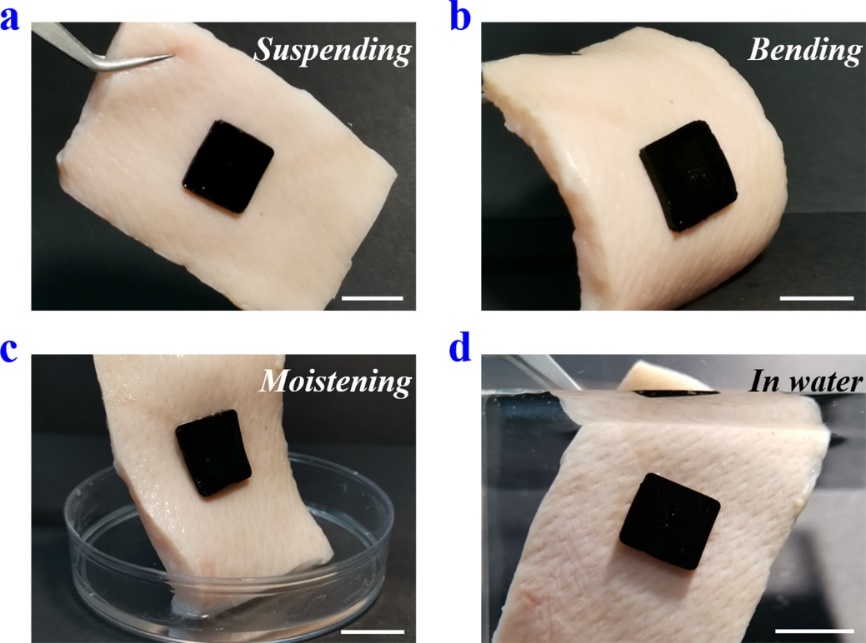


**Figure S3.** Digital images showing that multifunctional MNs adhered to the pork skin when they were suspending (a), bent (b), in a moist environment (c) and in water (d). The scale bars are all 1.5 cm.


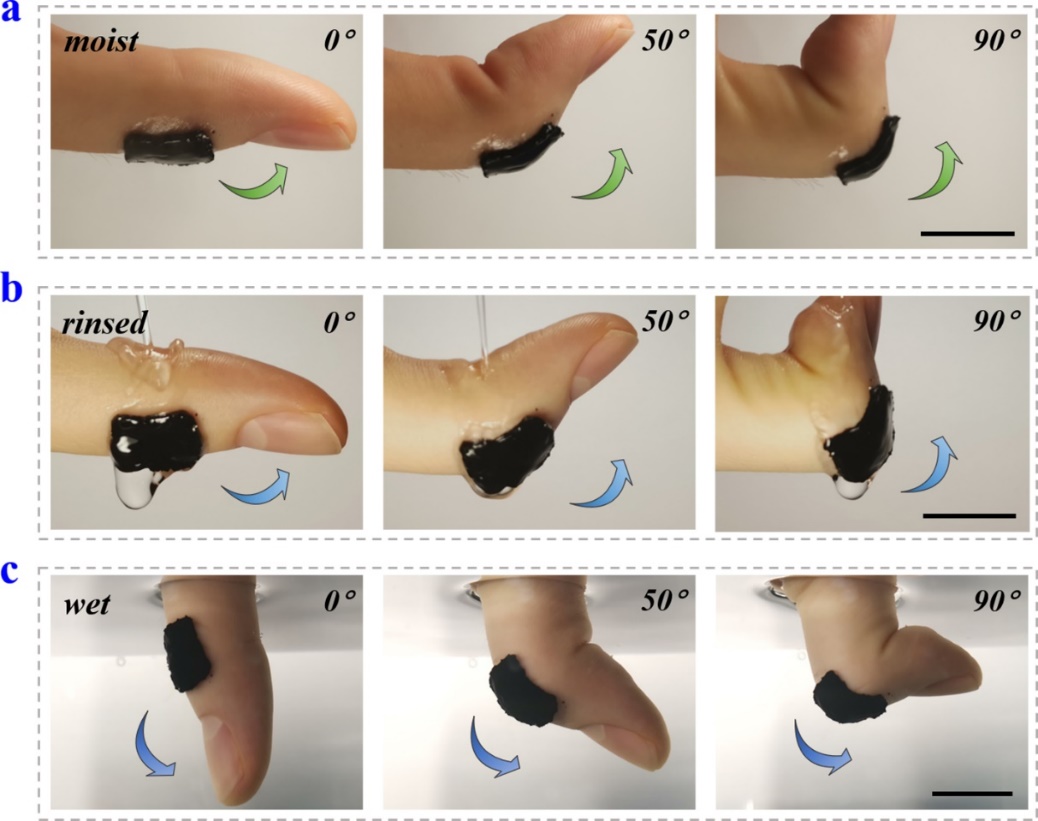


**Figure S4**. **Digital images showing that multifunctional MNs adhered to the knuckle of the thumb when they were bent from 0° to 90°.** (a) They were sprayed with water. (b) They were rinsed with water flow. (c) They were submerged under water. The scale bars are all 1.5 cm.


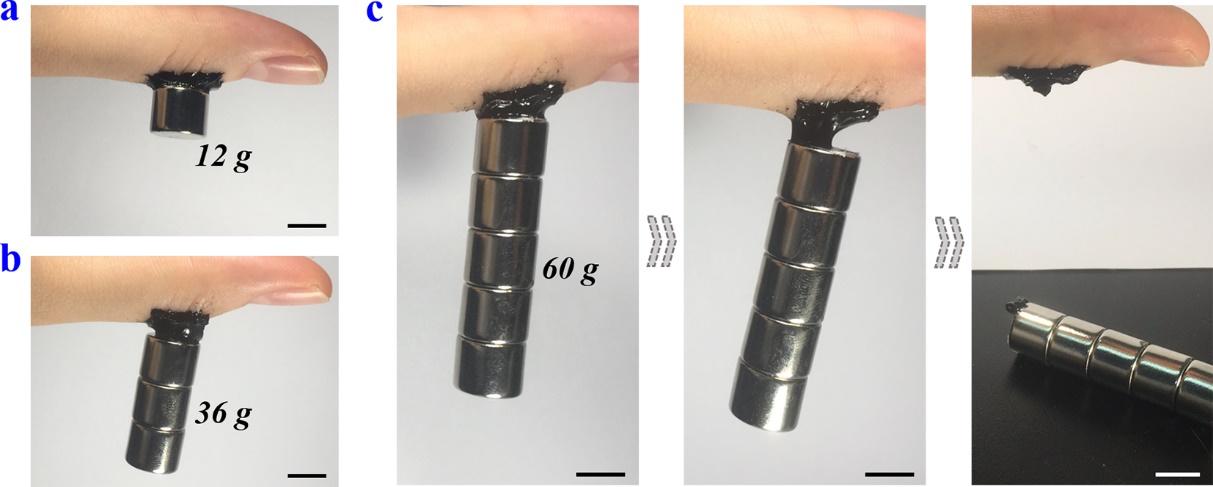


**Figure S5.** **Loading test of multifunctional MNs.** (a-b) The MNs adhered to the knuckle when the weights were 12 g (a) and 36 g (b). (c) Process of the MNs and weights falling off from the knuckle. Parts of the MNs remained on the knuckle. The scale bars are 7.5 mm.


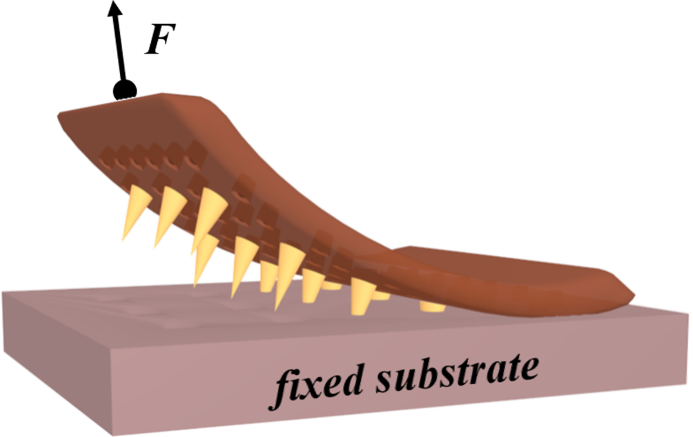


**Figure S6.** Schematic illustration of the peeling-off test process.


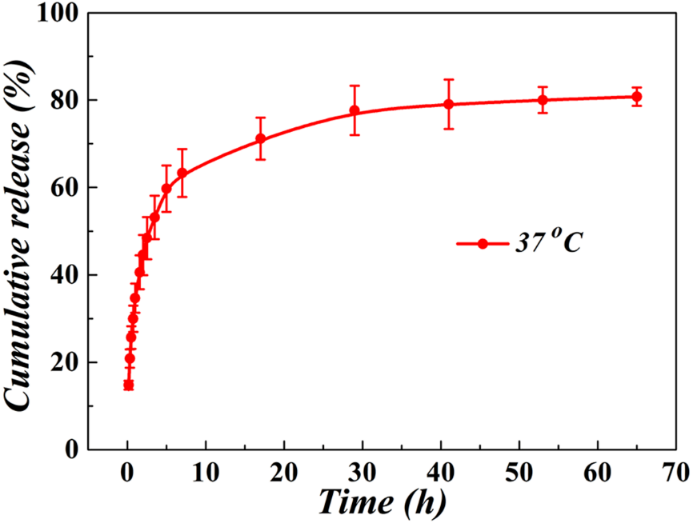


**Figure S7.** *In vitro* drug release of multifunctional MNs under 37 °C. RhB was chosen as a model drug.


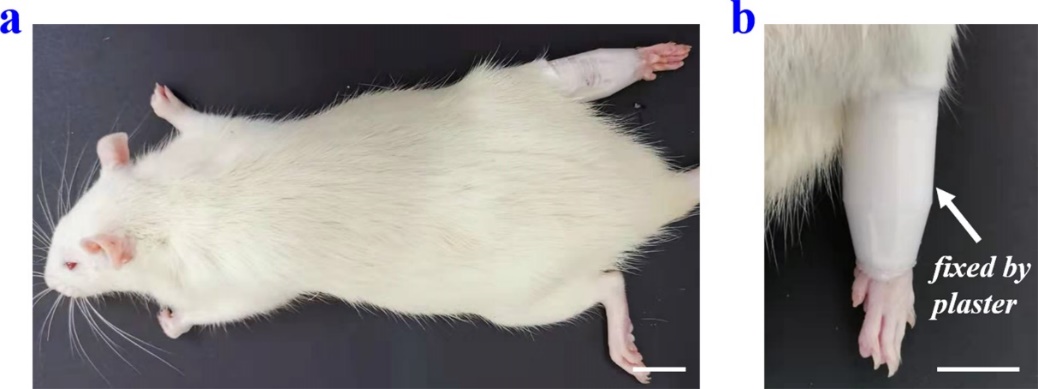


**Figure S8.** Digital images showing the establishment of the KOA rat model. The knee joints were fixed by the tubular plaster in the overextension position.


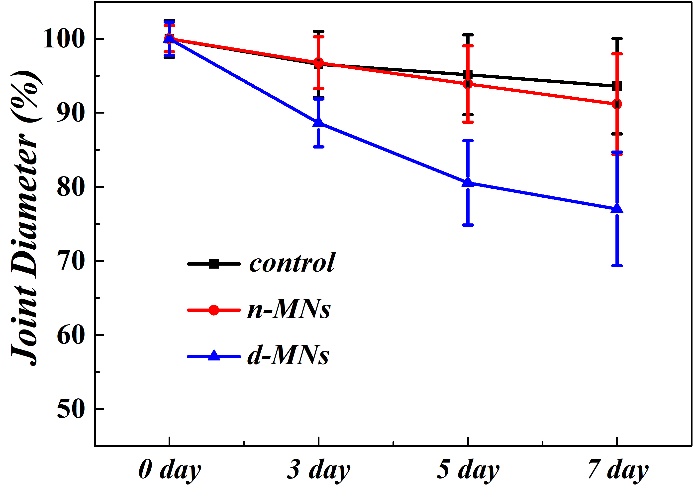


**Figure S9.** Changes in swollen degree of the rat knees. The swollen degree was reflected by the diameter of the joint.


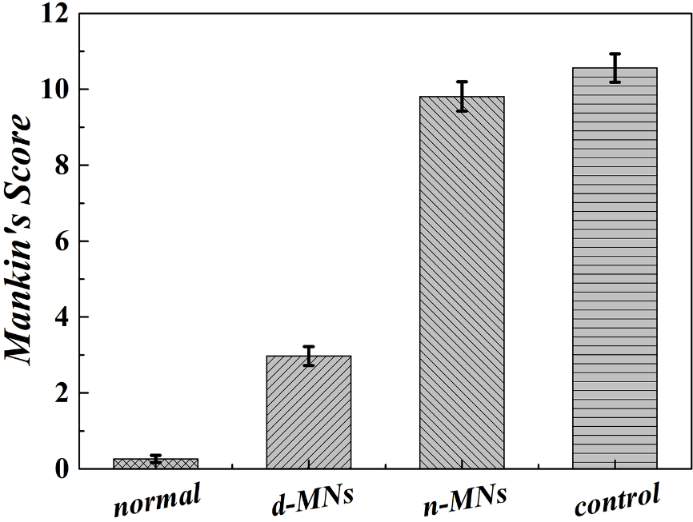


**Figure S10.** Grading of the severity of KOA lesion with the modified Mankin scoring system in different rat groups.
